# Differences of airborne and mural microorganisms in a 1,500-year-old Xu Xianxiu’s Tomb, Taiyuan, China

**DOI:** 10.3389/fmicb.2023.1253461

**Published:** 2023-10-25

**Authors:** Jiangyun Liu, Fasi Wu, Ting Xiang, Wenxia Ma, Dongpeng He, Qi Zhang, Wanfu Wang, Yulong Duan, Tian Tian, Huyuan Feng

**Affiliations:** ^1^School of Public Health, Lanzhou University, Lanzhou, Gansu, China; ^2^National Research Center for Conservation of Ancient Wall Paintings and Earthen Sites, Department of Conservation Research, Dunhuang Academy, Dunhuang, Gansu, China; ^3^Gansu Provincial Research Center for Conservation of Dunhuang Cultural Heritage, Dunhuang, Gansu, China; ^4^MOE Key Laboratory of Cell Activities and Stress Adaptations, Centre for Grassland Microbiome, School of Life Sciences, Lanzhou University, Lanzhou, Gansu, China; ^5^Northwest Institute of Eco-Environment and Resources, Chinese Academy of Sciences, Lanzhou, Gansu, China

**Keywords:** biodeterioration, aerosols, microorganisms, cultural heritage, murals

## Abstract

**Background:**

Microbial colonization represents one of the main threats to the conservation of subterranean cultural heritage sites. Recently, the microbial colonization on murals in tombs has gradually attracted attention.

**Methods:**

In this study, a total of 33 samples, including 27 aerosol samples and 6 mural painting samples, were collected from different sites of Xu Xianxiu’s Tomb and analyzed using culture-dependent methods. We compared the diversities of culturable bacteria and fungi isolated from the air and murals and explored the potential impacts of microorganisms on the biodeterioration of the murals.

**Results:**

Phylogenetic analyses revealed that the culturable bacteria belonged to Bacillus, Microbacterium, Lysobacter and Arthrobacter. And the most of fungal belonged to the Penicillium, Cladosporium and Aspergillus genera. The composition and structure of airborne bacteria and fungi outside the tomb were both significantly different from that inside the tomb. The variation trends of airborne bacterial and fungal concentrations at different sampling sites were remarkably similar. Bacillus frigoritolerans, Bacillus halotolerans, Bacillus safensis, Exiguobacterium mexicanum, Microbacterium trichothecenolyticum, and Micrococcus yunnanensis were bacterial species commonly isolated from both the mural and air environments. Fungal species commonly isolated from aerosol samples and mural painting samples were Alternaria alternata, Cladosporium cladosporioides, Penicillium brevicompactum, and Peyronellaea glomerata. The prediction of the ecological functions of the bacteria revealed that chemoheterotrophy or aerobic_chemoheterotrophy accounted for substantial relative proportions in all sample types.

**Conclusion:**

These results suggest that the aerosol circulation between the inside and outside environments of the tomb was weak and that the outside environment had yet to have an impact on the air microbial community inside the tomb. Selective colonization of microorganisms, which is mediated by interaction between microorganisms and special microenvironmental factors, is an important reason for the biodeterioration of murals.

## 1. Introduction

Cultural heritage artifacts suffer from permanent damage due to exposure to natural geological hazards, while wind erosion, water, light, and the slow loss of various mineral elements make the surface morphologies of cultural heritage artifacts change constantly (Zanardini et al., 2000; Pavlova et al., 2017; Wu et al., 2021b). Cultural heritage artifacts with various environment are inhabited by microorganisms. Although some microorganisms such as bacteria, fungi, and archaea have a positive or useful way in preserving cultural heritage, the impact of these microorganisms on deteriorating cultural heritage artifacts is undeniable (Cappitelli et al., 2020; Pyzik et al., 2021). And this biodeterioration effect is more pronounced when environmental conditions change, such as the temperature, relative humidity, pH and light (Michaelsen et al., 2006; Wu et al., 2022). Microbial colonization and deterioration are ubiquitous phenomena occurring extensively on mural paintings in catacombs or tombs. The substratum materials of mural paintings containing animal and plant origin materials that suffer easily from Microbial invasions when the environmental conditions are favorable for microbial growth (Ciferri, 1999; Pangallo et al., 2012). The biodeterioration caused by microorganisms accompanied by long-term colonization and large-scale outbreaks of different microbial groups has been one of the most important factors endangering the preservation of cultural heritage artifacts. Due to the importance of cultural heritage, it is important to investigate the activity of microbial communities in the environments of cultural heritage artifacts and the corresponding mechanism of biocorrosion (Urzi et al., 2016; Dominguez-Moñino et al., 2017; Zhang et al., 2023). Among the many kinds of cultural heritage artifacts, murals are undoubtedly the most special type that exist in a variety of cultural heritage sites. Moreover, the types of environments in which the microorganisms in the murals colonize are also varied, such as caves (Alonso et al., 2019; Genuite et al., 2021), grottoes (Wang et al., 2011, 2012; Duan et al., 2021), catacombs (Diaz-Herraiz et al., 2013; Tomassetti et al., 2017; Ma et al., 2020; De Leo et al., 2022), etc.

The protective principles of cultural heritage have been gradually shifting from salvage conservation to preventive conservation, which has been paid widespread attention for many years (Caneva et al., 2008; Dziurzynski et al., 2020; Wang et al., 2022). The purpose of microbial diversity research on murals is to predict the biodeterioration potential of murals and to provide basic data for supporting future conservation and restoration work. Actually, the microbial study on tombs has been going on for a long time with focus (e.g., bacteria, fungi, biocidal treatments, Aerobiology, microclimate, deterioration) changed constantly (Emoto, 1974; Agarossi et al., 1988; Monte et al., 1994; Mandrioli et al., 2003; Sprocati et al., 2008). Most previous studies have focused on visible biofilms and microbial speckles with different colors, including white, yellow, gray, black, and brown, that had formed on mural painting surfaces (Saiz-Jimenez et al., 2012; Vasanthakumar et al., 2013; Suphaphimol et al., 2022; Li et al., 2023). Not only are such samples easier to collect, but the microbes have already caused visible aesthetic damage to the murals (He et al., 2021; Ma et al., 2023), which is more likely to attract attention. However, from another point of view, when the accumulation of microorganisms has already caused irreversible damage to murals, this often means a delay in the research of cultural heritage. Therefore, it is urgent to conduct predictive research on microbial diversity in murals, which requires us to collect mural samples when the murals have not yet undergone serious biological corrosion. As early as 1988, Arai, H. et al. isolated several fungi and bacteria from the blackening of a blue pigment on the ceiling and plaster in the tomb (Arai, 1988). A seminal study in which the researchers had directly scraped samples of mural pigments in the Altamira cave and analyzed the microbial diversity in the samples was carried out by Schabereiter-Gurtner et al. (2002) to predict the potential microbial threats in 2002. This is the first time that the researchers were allowed to directly collect murals for microbial analysis in the cave. With the maturity and wide application of environmental monitoring technology in recent years, the environmental characteristics of tombs had gradually become an important part of the research (Huang et al., 2017; Caneva et al., 2020). Moreover, the influence of airborne microbial communities on murals has gradually been recognized, and the survival status of microorganisms is an important indicator of the risk of microbial deterioration of murals (Saiz-Jimenez and Gonzalez, 2007; Pangallo et al., 2012; Martin-Sanchez et al., 2014). Aerobiology research applied to the conservation of cultural heritage aims to assess the risk of airborne microorganisms on materials of artefacts (Mandrioli et al., 2003). Airborne microorganisms with a wide range of transmission can adapt to various types of ecological environments, and their impact on cultural heritage cannot be ignored (Leplat et al., 2020; Duan et al., 2021). Therefore, it is necessary to study the microorganisms in the air environments around cultural heritage sites. The aerosols in the atmosphere contain a wide variety of material components from a wide variety of sources, including vegetation and soil (Lighthart and Shaffer, 1994), marine inputs (Feltracco et al., 2021), animal farming (Zucker et al., 2000; Wilson et al., 2002), and transportation (Ozga et al., 2014; Gaylarde et al., 2018). Previous studies have shown that *Bacillus*, *Micrococcus*, *Staphylococcus*, and *Pseudomonas* are often present in the air (Lee et al., 2021; Mishra et al., 2021), but a few research has been done about the microorganisms in the air environments surrounding murals in tombs.

Xu Xianxiu’s Tomb is located in Taiyuan city, Shanxi Province, China. In the tomb, there are nearly 330 square meters of large-scale mural figure paintings painted inside the chamber. Because these murals are large in size and difficult to move and store, maintaining their integrity becomes one of the difficulties in conservation work. At present, the wall paintings in the dromos and the chamber suffer from serious damage, such as efflorescence and collapse. Due to the environment, this cultural heritage site is in urgent need of repair, but there are still few studies on the microorganisms inside that tomb, and it is difficult to provide a sufficient scientific basis and data reference for the protection of the murals.

The objectives of the present study were to (1) elucidate the distribution patterns of bacterial and fungal communities in different growing environments through identifying the culturable bacteria and fungi isolated from the air and deteriorated murals and comparing the bacterial and fungal diversity and community compositions; (2) assess the impacts of the microenvironmental conditions on the community distribution patterns; (3) attempt to explore the sources of bacteria and fungi in the aerosol and murals and their potential functions; and (4) explore the relationships among bacteria and fungi in this delicate ecosystems.

## 2. Materials and methods

### 2.1. Site description and sampling

**Figure 1:**
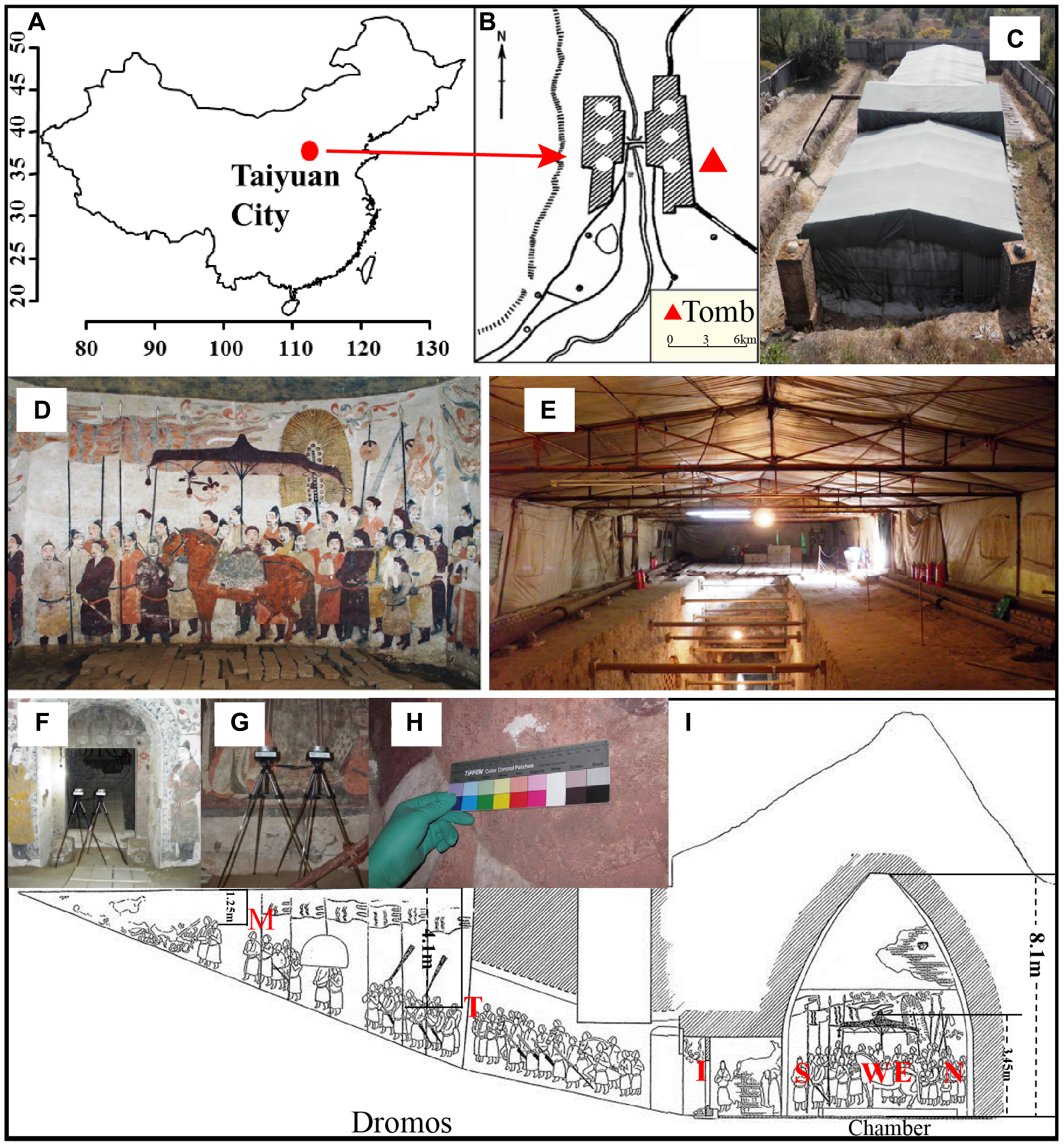
Images of Xu Xianxiu’s Tomb. **(A,B)** Study area and location of Xu Xianxiu’s Tomb; **(C)** outdoor view of Xu Xianxiu’s Tomb; **(D,E)** indoor view of Xu Xianxiu’s Tomb. Relative positions of the aerosol sampling sites in Xu Xianxiu’s Tomb: **(F,G)** the samples of aerosols were collected near mural paintings with a Buck Bio-Culture™ sampler. Relative positions of the mural sampling sites in Xu Xianxiu’s Tomb: **(H)** the sampling sites of the mural paintings in Xu Xianxiu’s Tomb. **(I)** Schematic diagram of the locations of the sampling sites.

Xu Xianxiu’s Tomb is located in Taiyuan city (37′50′11.8 N; 112′36′42.2′E; altitude at 900 m). It lies in a typical north temperate zone and has a continental monsoon climate with a mean annual temperature of 9.5°C, a mean annual precipitation of 456 mm. The rainfall is concentrated in summer and autumn, and winter and spring are dry and windy. The microenvironment in the indoor tomb varies with the seasons. The temperature is 2–15°C and relative humidity is 60–100% in the indoor tomb. The temperatures in the corridor and dromos are 3–22°C, which is higher than that in the indoor tomb. The relative humidity outside the tomb varied between 20 and 100%, and the temperature is −7–35°C. To keep track of changes in temperature and relative humidity, we placed data loggers (Onset HOBO, USA) in different locations to monitor temperature and relative humidity once every half an hour (Wu et al., 2021a). Xu Xianxiu’s Tomb was rated as one of the top ten new archeological discoveries in China in 2002. The cultural heritage protection workers carried out urgent conservation work, such as building a roof to cover the main structure of the tomb, in October 2002 (Figures 1A–E).

The aerosol and murals sample were collected from different sites of Xu Xianxiu’s Tomb, which was never opened to the public during the time from excavation to sampling except when only a few staff carried out urgent partial repairs of the murals. Therefore, it is believed that the tomb was not disturbed excessively by humans before the time of sampling. The collected samples can reflect the real microorganism community levels in the tomb.

We used a Buck Bio-Culture™ sampler approximately 0.5 m from the surface of the murals and approximately 1.5 m from the ground to collect air samples at a flow rate of 30 L/min for 5 min. Before sampling, we used 75% ethanol to disinfect the sampler and then placed a Petri dish with medium [Reasoner’s 2A Agar (R2A) medium/1 L: yeast 0.5 g, tryptone 0.5 g, casein hydrolysate 0.5 g, glucose 0.5 g, starch 0.5 g, HK_2_PO_4_ 0.3 g, MgSO_4_·7H_2_O 0.05 g, sodium pyruvate 0.3 g, agar 15.0 g, pH 7.2; Potato Dextrose Agar (PDA) medium/1 L: potato juice 1,000 ml, glucose 20 g, agar 15 g] in the sampler to collect aerosol samples. A Petri dish 90 mm in diameter is suitable for the size of the sampler. All of the solid media with flat surfaces and uniform thicknesses were freshly prepared at the same time. After confirming that there was no microbial contamination during the medium preparation process through the empty culture experiment at 37°C for 48 h, the medium was uniformly sealed with parafilm and brought into the sampling sites for sample collection.

Air samples (27 samples in all) were collected from three locations: (1) Near the north wall (N), west wall (W), south wall (S), east wall (E), and upper east wall (ES) of the inner chamber (CH); (2) The tomb passage (I) and the east wall (T) and west wall (M) of the dromos (DR); and (3) The outdoors (OD) near the entrance of the tomb (Figure 1I). Three air samples were collected at each site under identical conditions as parallel experiment for each sample. After the Petri dish in the sampler was removed, 75% ethanol was used to disinfect the sampler, and the sample was placed in another Petri dish after the ethanol was completely volatilized. The Petri dishes with aerosol particles were sealed with parafilm and transported to the laboratory, where they were incubated at room temperature for 72 h before subsequent analysis.

The mural samples were collected from different sites of the mural layers in Xu Xianxiu’s Tomb. Except for the sampling sites of OD and ES, the sampling sites of the mural samples and the air samples were in one-to-one correspondence (Figure 1I). Murals samples (9 samples in all, namely, 1#-9#) were collected from two locations: (1) Near the north wall (N, 3#,7 #), west wall (W, 4#), south wall (S, 5#), east wall (E, 1#, 2#, 6#) of the inner chamber (CH); (2) The tomb passage (I, 8#) and the east wall (T, 9#) of the dromos (DR). The specific samples collected are shown in Table 1.

**Table 1:** Sampling sites of the air and murals in Xu Xianxiu’s Tomb.

Site name	Site location	Air samples	Murals samples
N	CH	3	2 (3#,7 #)
W	CH	3	1 (4#)
S	CH	3	1 (5#)
E	CH	3	3 (1#, 2#, 6#)
ES	CH	3	–
I	DR	3	1 (8#)
T	DR	3	1 (9#)
M	DR	3	–
OD	–	3	–

Mural samples were collected by scraping the paint layer on the surface of the mural with a sterile scalpel. In accordance with the relevant regulations of cultural heritage protection, we selected the damaged parts of the murals as the sampling sites as much as possible to minimize the mechanical damage on the murals. We were accompanied by staff members of the Dunhuang Academy and the Taiyuan Northern Qi Mural Museum during the whole sampling process. To avoid the contamination of the samples, the researchers wearing sterile gloves scraped the samples into 2 ml sterile centrifuge tubes, sealed and transported the sterile centrifuge tubes to the laboratory.

### 2.2. Cultivation, isolation and identification of bacteria and fungi

The culture process of bacteria is as follows: firstly, 20 mg of each mural sample was weighed and placed into a 2 ml centrifuge tube containing sterile physiological saline, and then some glass beads were added and vortex oscillation was applied for 15 min. Then, 1 ml of suspension was inoculated into a 50 ml Peptone Yeast Extract Glucose Vitamin (PYGV) liquid medium in a culture flask (Staley, 1968) and incubated at 20°C and 150 rpm for 15 days. Nine parallel samples from 100 μl of the cultured bacterial suspension were spread evenly on the R2A solid medium after being diluted, and these plates were cultivated at room temperature for 2–7 days until no new bacterial colonies appeared. And the culture process of fungi is as follows: 20 mg of each mural sample was weighed and placed into a 1.5 ml centrifuge tube containing sterile physiological saline, and then some glass beads were added and vortex oscillation was applied for 10 min. Then, 800 μl of suspension was inoculated into a 50 ml PYGV liquid medium in a culture flask and incubated at 25°C and 150 rpm for 25 days. After microbial colonies had grown on all media, they were simply classified by distinguishing their external morphological characteristics, and the microbial colonies in each Petri dish were counted according to the classification. The number of culturable microbia in the air samples is expressed by colony forming units (CFU) per cubic meter of air, namely, CFU/m^3^. The number of culturable microbia in mural samples is expressed in CFU/gram of mural samples, that is, CFU/g. After the preliminary typing of the bacterial colonies by basic indicators such as shape, color, and smoothness, beef extract peptone medium (NA medium/L: peptone 10 g, beef extract 3 g, NaCl 5 g, agar 15 g, pH 7.2) was used to isolate and purify single bacterial colonies with different phenotypes. The single colonies were inoculated into NA solid medium by the continuous streaking method and incubated at 37°C for 48 h. Finally, the bacterial DNA was directly extracted from the pure cultured strains. According to the morphology of fungal colonies, spores, mycelia and so on, the preliminary identification and classification were carried out, and then the PDA medium was used to separate, purify and culture different fungal colonies until a pure cultured strains appeared. Fungal DNA was extracted by liquid nitrogen grinding and SDS method, which was slightly modified on the basis of previous studies (Moller et al., 1992). During physical wall breaking, 5 μl of lyticase (20 mg/mL) was added while protease K was added for incubation.

The bacterial 16S rRNA gene was amplified using the primer set 27F, 5’-AGAGTTTGATCCTGGCTCAG-3′, and 1492R, 5’-TACGGCTACCTTGTTACGACTT-3′; the fungal ITS1 region was amplified using the primers ITS1F, 5′- TCCGTAGGTGAA CCTGCGG-3′ and ITS4, 5′-TCCTCCGCTTATTGATATGC-3′. Polymerase chain reaction (PCR) experiments were carried out with a 25 μl reaction volume containing 1 unit of Taq DNA polymerase (Tiangen Co., Beijing, China), 0.2 mM dNTPs, 2.5 μl of 10-fold Taq DNA polymerase buffer, 2.5 mM MgCl_2_, 0.2 μM of each primer, and 2.5 μl of bacterial genomic DNA template (*ca.* 10 ng). The PCR program for the 16S rRNA gene included an initial denaturation at 94°C for 3 min; 30 cycles at 94°C for 1 min, annealing at 58°C for 1 min, and extension at 72°C for 1.5 min; and a final extension for 10 min at 72°C. The following PCR program for the 18S rRNA gene was used: initial denaturation at 94°C for 5 min; 94°C for 1 min, 30 cycles at 58°C for 1 min, and extension at 72°C for 1 min; and extension for 10 min at 72°C; and 4°C for 10 min. PCR products were detected by electrophoresis in 1% agarose gels, and those with suitable fragment sizes and clear single bands were used for restriction fragment length polymorphism (RFLP) analysis. In the RFLP analysis, PCR products after digestion by *Csp6* (BsuR I for fungi) and *HinfI* (MBI, Fermentas) at 37°C for 3.5 h (4 h for fungi) were detected by 3.0% (2.5% for fungi) agarose gel electrophoresis. Digestion products with the same spectrum types were grouped into a single category, and those with different spectrum types were identified by sequencing.

The PCR products were purified using a Gel Extraction Kit (Tiangen Co., Beijing, China). Cloning was performed with the pGEM-T Vector System (Tiangen Co., Beijing, China) following the manufacturer’s protocol. The ligation products were subsequently transformed into *Escherichia coli* DH5α cells for blue-white screening. Transformants were plated onto LB medium containing ampicillin, IPTG (40 mM) and X-Gal (2%, w/v). The positive clones were dissolved with 10 μl sterile dd H2O, and the DNA template obtained by the freeze–thaw method was identified by PCR amplification with pGEM-T vector-specific primers (T7/SP6) (Maior Biotech Co., Ltd., Shanghai, China). The PCR amplification procedure was as follows: an initial denaturation at 94°C for 5 min; 30 cycles at 94°C for 1 min, annealing at 58°C for 1 min, and extension at 72°C for 1.5 min; and a final extension for 10 min at 72°C.

The obtained DNA sequences were edited with CONTIGEXPRESS (InforMax, Inc., MD), and the chimaeras were identified and removed using the chimera check. The most similar sequences were extracted from the GenBank database^1^ and the EzTaxon-e database. A phylogenetic neighbor-joining tree, including the obtained sequences and their closest relatives, was constructed using the Jukes-Cantor model in MEGA software 5.0. The sequences retrieved from this study can be accessed under NCBI accession numbers KC429584–KC429650 for the culturable mural bacteria, JX094156-JX094180 for the culturable airborne bacteria, KJ780796–KJ780822 for the culturable mural fungi, JX136715–JX136749 for the culturable airborne fungi.

### 2.3. Processing and analysis of the sequencing data

All raw experimental data were tested for normality and equal variance before analysis. The differences in the bacterial concentration and diversity index between different sites were tested using one-way ANOVA or t test. The above statistical analyses were performed using SPSS 16.0 software (SPSS Inc., Chicago, USA). Shannon’s diversity index (*H*′) was determined using Paeontological Statistics (PAST) 2.03 software (Hammer et al., 2001). Principal component analysis (PCA) was generated in R^2^ (version 4.0.4). The functional taxa of the cultural bacteria were annotated with the python script (collapse_table.py) in the Python 3 environment by FAPROTAX database (version 1.2.6^3^, Accessed on 19 May 2023) (Zhang et al., 2023).

## 3. Results

### 3.1. Diversity and community composition of the culturable microorganisms isolated from aerosols and mural

**Figure 2:**
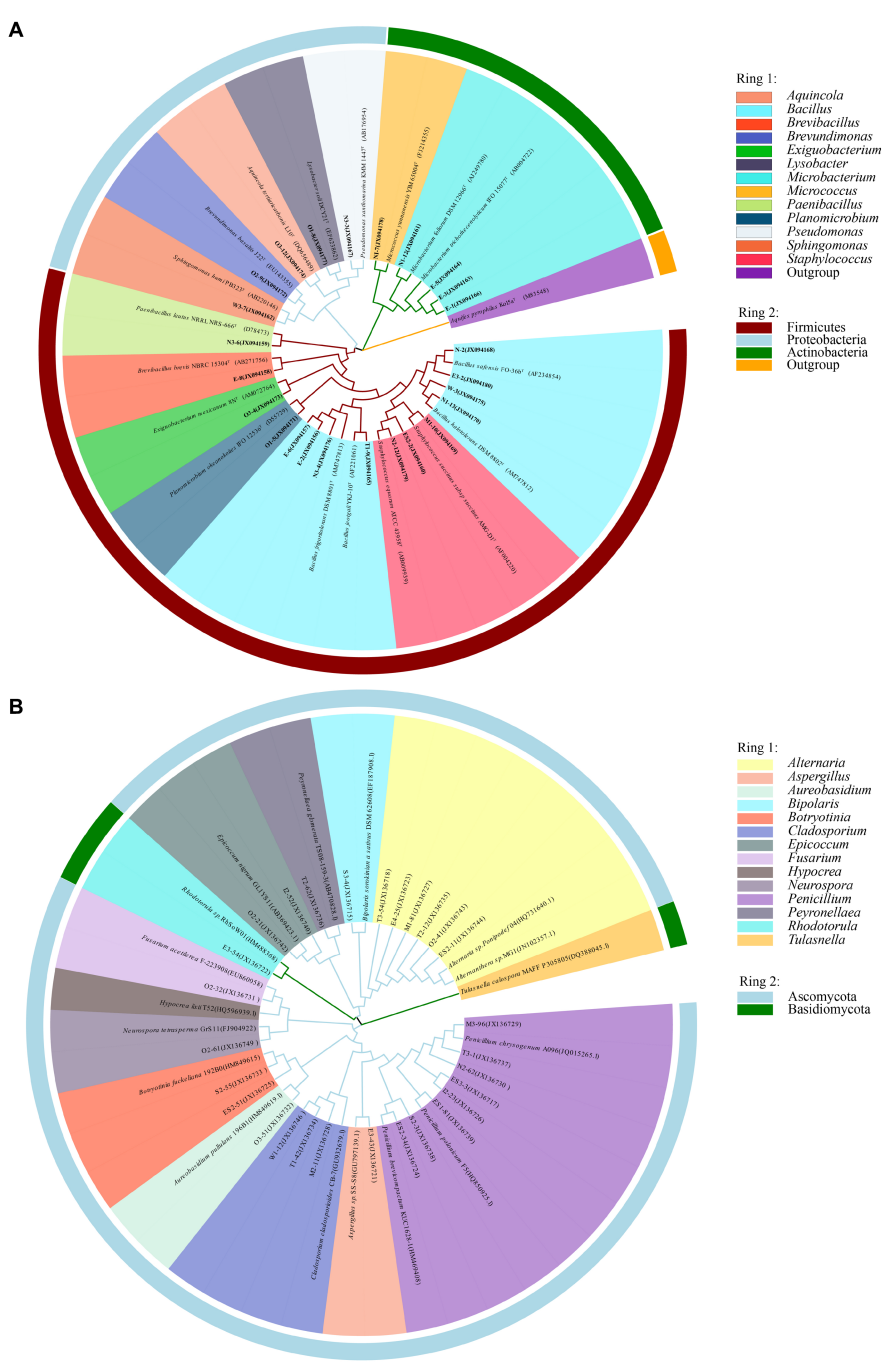
Phylogenetic tree of gene sequences derived from the **(A)** airborne bacteria and **(B)** airborne fungi of the Xu Xianxiu’s Tomb.

**Figure 3:**
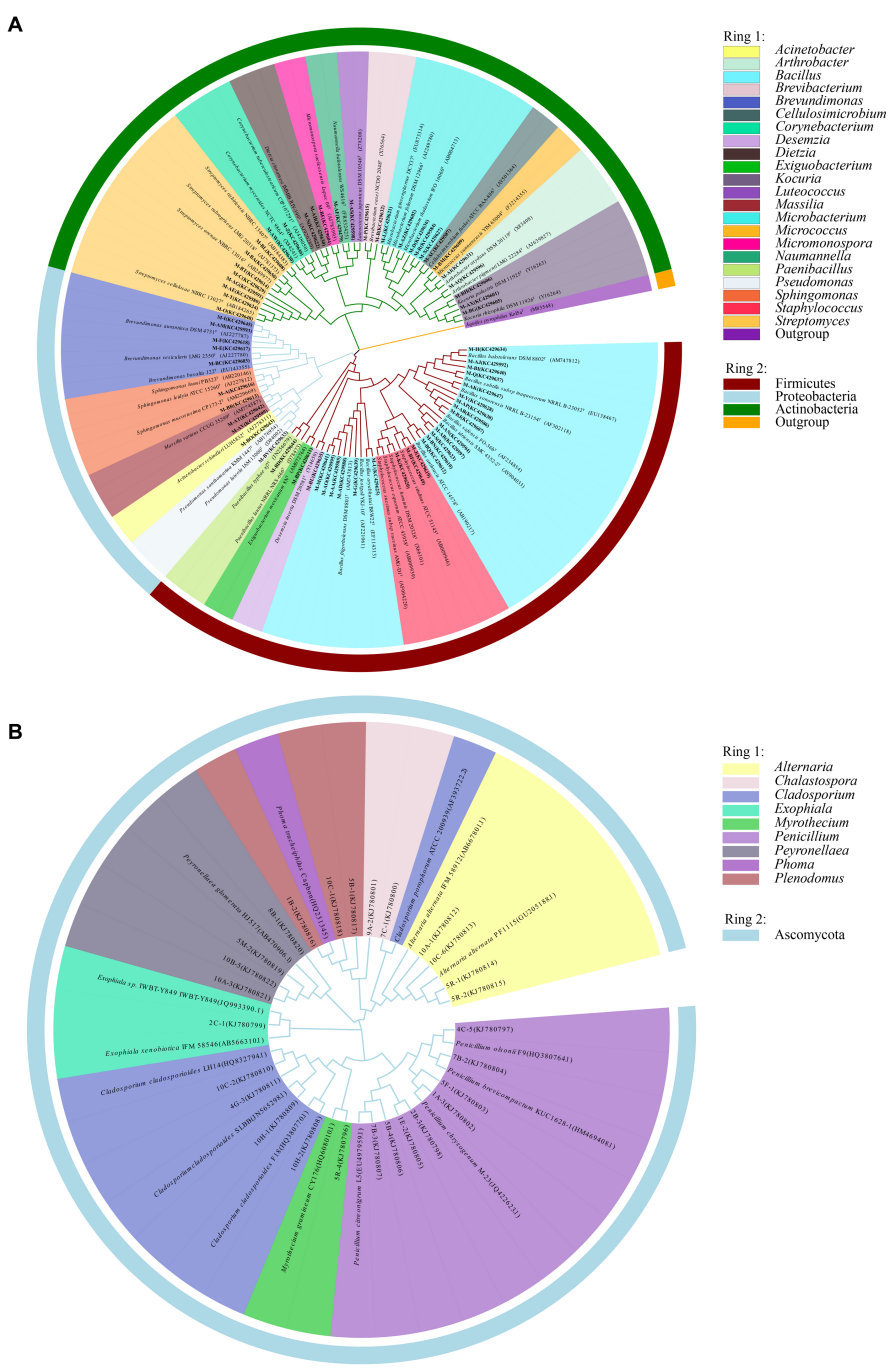
Phylogenetic tree of gene sequences derived from the **(A)** mural bacteria and **(B)** mural fungi of the Xu Xianxiu’s Tomb.

Information on the microbial communities was obtained by PCR amplification and the extraction of the genomic DNA from samples. All the bacterial sequences isolated from the air samples were classified into 17 different species, with 13 genera and 3 phyla affiliated with them (Figure 2A). And the bacterial sequences isolated from the mural samples were classified into 48 different species, with 22 genera and 3 phyla affiliated with them (Figure 3A). Among them, the *Naumannella* bacterium isolated from the mural in the CH site has been confirmed as a new species by physiological, biochemical and molecular identification (Tian et al., 2017). All the fungal sequences isolated from the air samples were classified into 14 genera and 2 phyla (Figure 2B). And the fungal sequences isolated from the mural samples were classified into 9 genera and 1 phylum (Figure 3B). In addition, a small amount of *Rhodotorula* of Basidiomycota was isolated and identified only from the air samples. Air samples and mural samples shared three genera: *Penicillium*, *Cladosporium* and *Alternaria*, and these three genera were dominant in the 2 types of samples.

**Figure 4:**
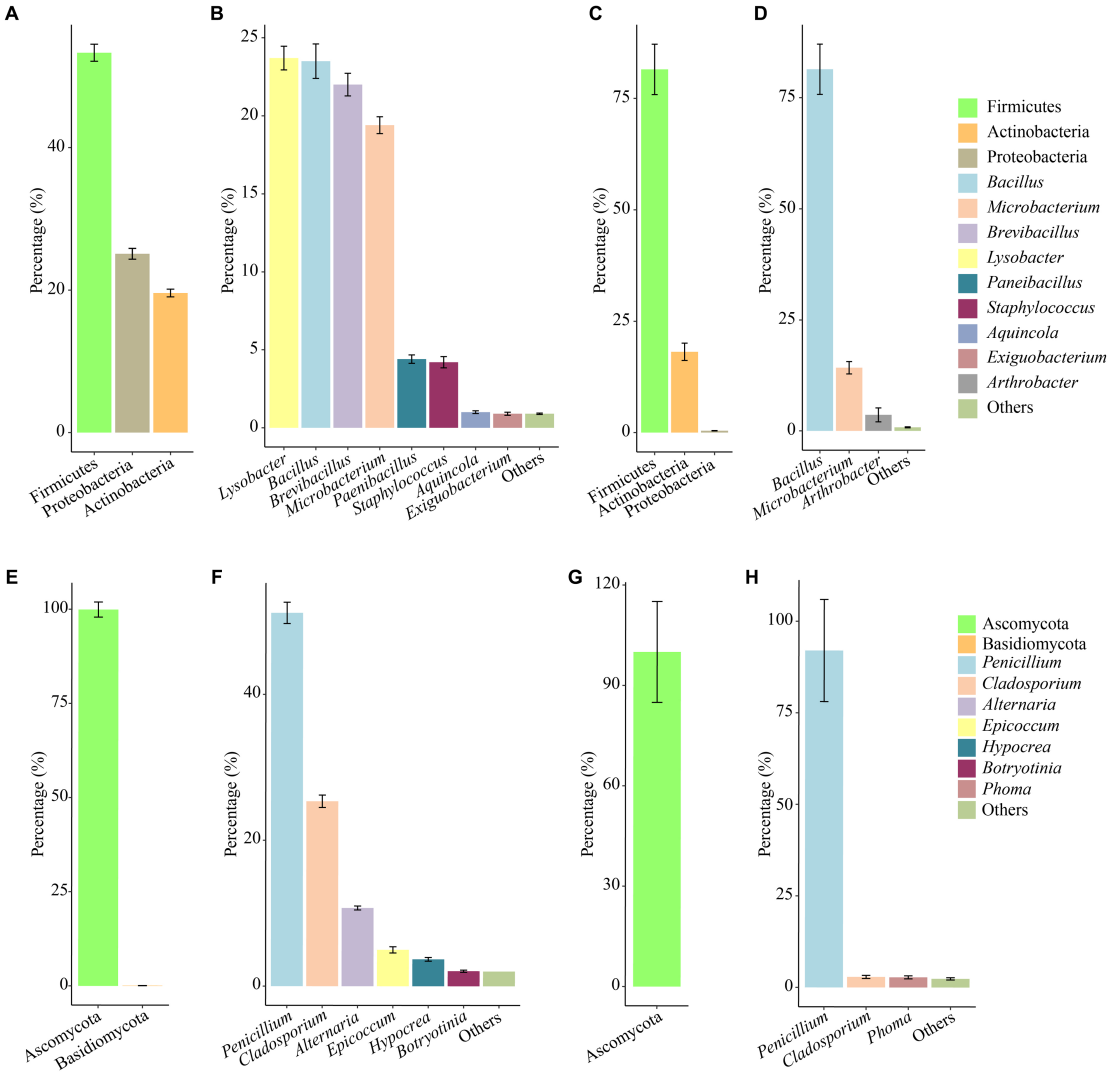
Abundance proportions of the predominant culturable bacteria and fungi. Bacteria at the phylum and genus levels isolated from the aerosol **(A,B)** inside and outside Xu Xianxiu’s Tomb and the murals **(C,D)** in Xu Xianxiu’s Tomb; fingi at the phylum and genus levels isolated from the aerosol **(E,F)** inside and outside Xu Xianxiu’s Tomb and the murals **(G,H)** in Xu Xianxiu’s Tomb.

In the culturable bacterial community of air samples, the concentration of Firmicutes was the highest, followed by Proteobacteria, and Actinobacteria was the lowest, accounting for 55.3, 25.1 and 19.6%, respectively. At the genus level, *Lysobacter*, *Bacillus*, *Brevibacillus*, and *Microbacterium* were the dominant genera, accounting for 23.7, 23.5, 22.0, and 19.4%, respectively (Figures 4A,B). In the culturable bacterial community of the mural samples, Firmicutes was the major bacterial phylum and accounted for 81.5% of the entire community. At the genus level, *Bacillus* was the most widely distributed in the mural samples and was the dominant genus, accounting for 81.4% of the culturable bacterial community (Figures 4C,D). For fungal communities of air samples, Ascomycota accounted for more than 99.91% of the microbial communities, in which Basidiomycota accounted for only 0.09% (Figure 4E). *Penicillium*, *Cladosporium*, *Alternaria*, *Epicoccum*, *Hypocrea*, *Botryotinia* were the dominant genera (Figure 4F). However, Ascomycota was the only phylum in the fungal community of mural, and the dominant genus *Penicillium* accounts for more than 92.01% (Figures 4G,H).

### 3.2. Concentrations and community structures of the culturable airborne and mural microorganisms

**Figure 5:**
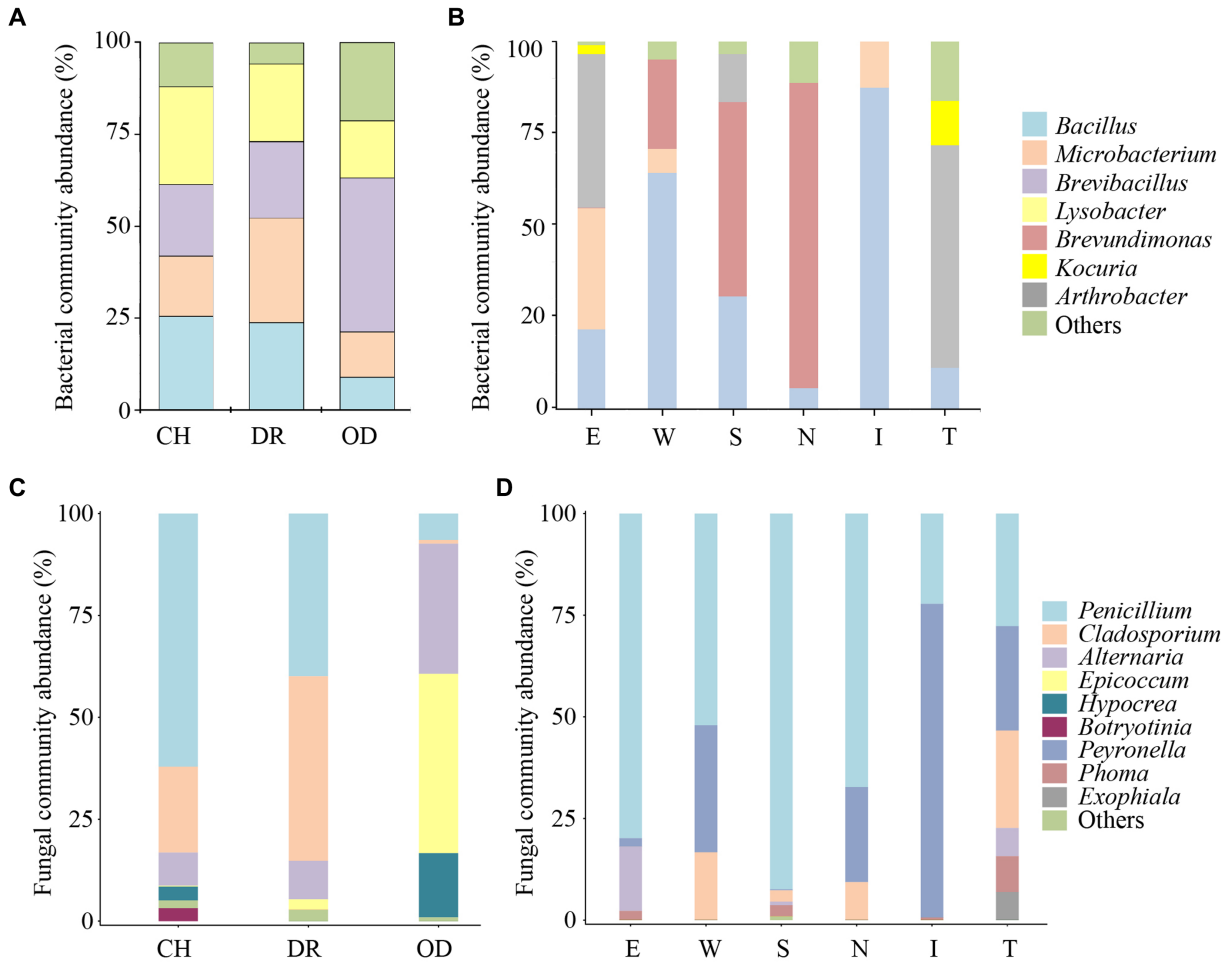
Proportions (≥10% for bacteria, ≥1% for fungi) of the predominant bacteria and fungi isolated from the air environment and murals at different locations inside and outside Xu Xianxiu’s Tomb. Abundance of the predominant culturable bacteria at the genus level isolated from the air environment **(A)** and murals **(B)** at different locations in Xu Xianxiu’s Tomb. Abundance of the predominant culturable fungi at the genus level isolated from the air environment **(C)** and murals **(D)** at different locations in Xu Xianxiu’s Tomb. CH, DR, and OD denote the chamber, dromos and outdoors, respectively.

The dominant air-culturable bacteria isolated from the CH, DR and OD sites were *Lysobacter*, *Microbacterium*, and *Brevibacillus*, accounting for 26.6, 28.5 and 41.8%, respectively (Figure 5A). There were significant differences in bacterial community composition at the OD site compared to the those at CH and DR sites. For example, the genera *Bacillus*, *Microbacterium*, and *Lysobacter* all had a high distribution in CH and DR, but had a low distribution in OD. The dominant culturable bacteria on the mural painting were different with different sampling sites, such as *Arthrobacter* (41.9 and 60.7%, respectively) for E and T sites, *Bacillus* (64.3 and 87.4%, respectively) for W and I site, *Brevundimonas* (52.9 and 83.3%, respectively) for S and N sites (Figure 5B). The most abundant genera of air-culturable fungi isolated from the CH, DR and OD sites were *Penicillium*, *Cladosporium* and *Epicoccum*, accounting for 62.1, 45.3, and 44.0%, respectively (Figure 5C). *Penicillium* was the dominant genus in all mural sampling sites, except for site I, in which *Peyronella* accounted for more than 77.1% (Figure 5D).

**Figure 6:**
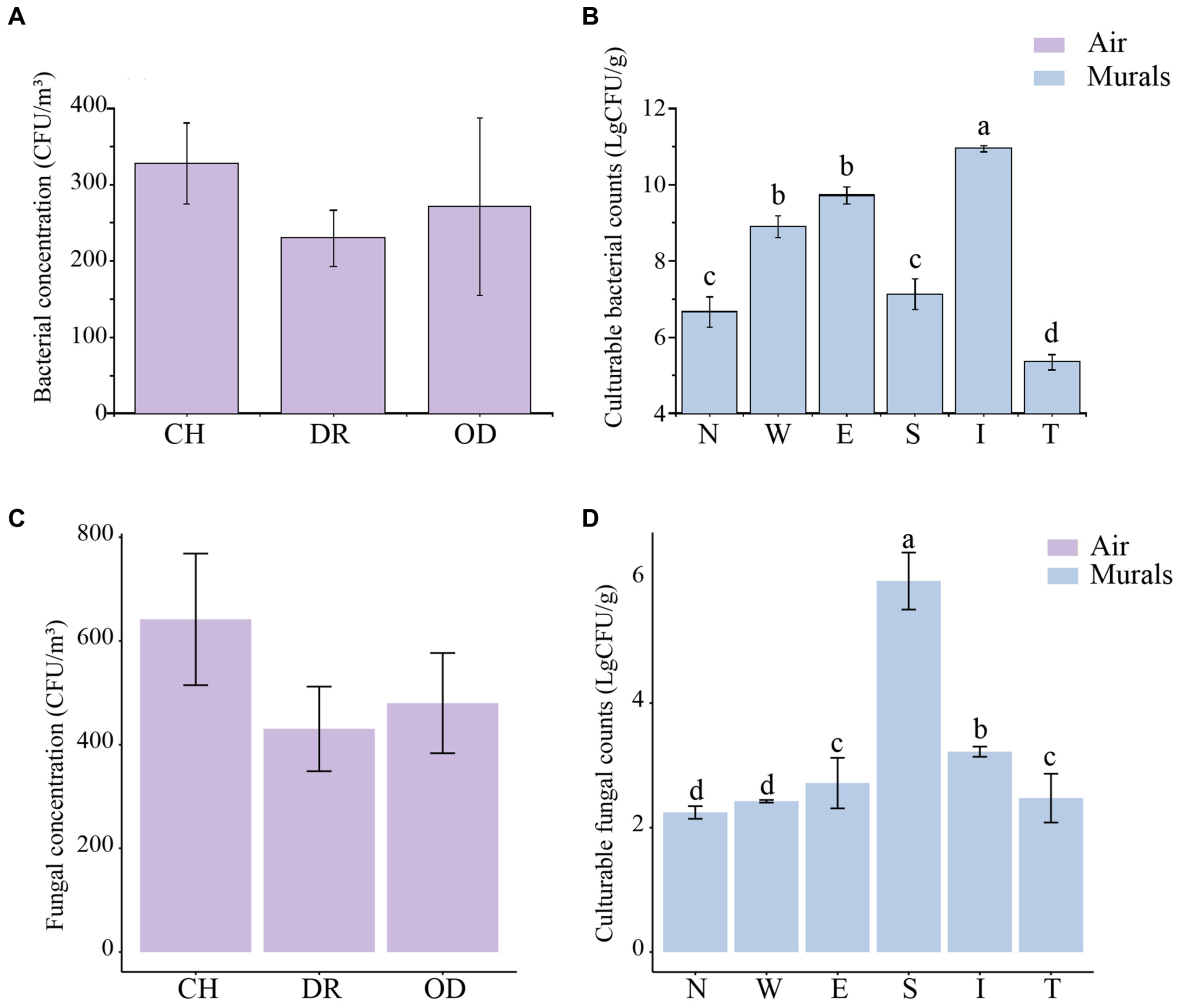
**(A,C)** Differences in airborne bacterial and fungal concentrations (CFU/m^3^) at different locations inside and outside Xu Xianxiu’s Tomb. CH, DR, and OD denote the chamber, dromos and outdoors, respectively. Error bars represent the mean ± the standard error. Significant differences among columns were determined using Tukey’s honestly significant difference (HSD) test (*p* ≤ 0.05). **(B,D)** Differences in culturable cell counts (LgCFU/g) of bacteria and fungi isolated from murals at different locations in Xu Xianxiu’s Tomb. Error bars represent means ± the standard error. Significant differences across columns were determined using the Tukey’s honestly significant difference (HSD) test (*p* ≤ 0.05) and are indicated by dissimilar letters (a-d) above the bars.

The airborne bacterial concentrations at sites CH, DR, and OD were 3.28 ± 0.53 × 10^2^ CFU/m^3^, 2.30 ± 0.36 × 10^2^ CFU/m^3^ and 2.71 ± 1.16 × 10^2^ CFU/m^3^, respectively. One-Way ANOVA showed that there was no significant difference in the airborne bacterial concentrations among the three sites (*F* = 0.858, *p* = 0.437; Figure 6A). The culturable cell counts of bacteria isolated from the mural samples at different sites in the tomb was generally higher. In the mural samples of the chamber, the culturable cell counts of bacteria at sites N, W, E, and S were 6.66 ± 0.39 (LgCFU/g) and 8.90 ± 0.28 (LgCFU/g), 9.72 ± 0.22 (LgCFU/g) and 7.12 ± 0.39 (LgCFU/g), respectively. In the dromos, the culturable cell count of bacteria at site I was 10.95 ± 0.08 (LgCFU/g), while that at site T was 5.35 ± 0.20 (LgCFU/g). The results of one-way ANOVA showed that the numbers of bacteria at sites E and W were significantly higher than those at sites N and S, but there was no difference between those of sites E and W. In addition, the numbers of bacteria at sites S and N were also not significantly different. The number of bacteria at site I of the dromos was significantly higher than that at site T, and there was a significant difference between the numbers of bacteria at sites I and T and those of the other sites in the tomb (*F* = 62.3667, *p* < 0.001; Figure 6B). The airborne fungal concentrations at sites CH, DR, and OD were 6.42 ± 1.27 × 10^2^ CFU/m^3^, 4.30 ± 0.82 × 10^2^ CFU/m^3^ and 4.80 ± 0.97 × 10^2^ CFU/m^3^, respectively (Figure 6C). In the mural samples of the chamber, the culturable cell counts of fungi at sites N, W, E, and S were 2.24 ± 0.10 (LgCFU/g), 2.42 ± 0.02 (LgCFU/g), 2.72 ± 0.41 (LgCFU/g) and 5.96 ± 0.46 (LgCFU/g), respectively. In the dromos, the culturable cell count of fungi at site I was 3.22 ± 0.08 (LgCFU/g), while that at site T was 2.48 ± 0.39 (LgCFU/g) (Figure 6D). It was worth noting that the variation trends of airborne bacterial and fungal concentrations at different sampling sites were remarkably similar.

**Figure 7:**
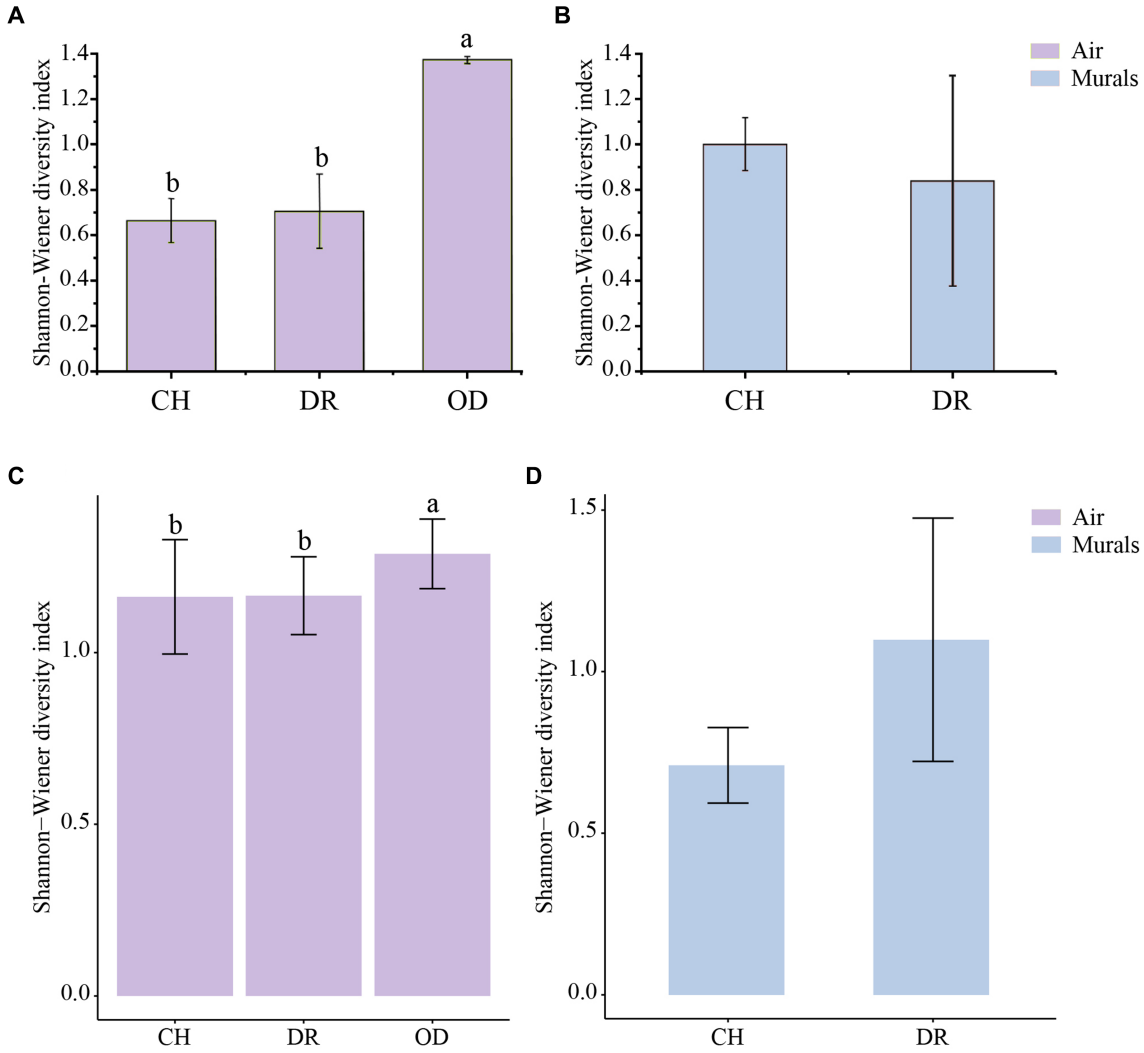
**(A,C)** Differences in the Shannon–Wiener diversity indices of the airborne bacterial and fungal community at different locations inside and outside Xu Xianxiu’s Tomb. CH, DR, and OD denote chamber, dromos and outdoors, respectively. Error bars represent the mean ± the standard error. Significant differences across columns were determined using Tukey’s honestly significant difference (HSD) test (*p* ≤ 0.05) and are indicated by dissimilar letters above the bars. **(B,D)** Differences in the Shannon–Wiener diversity indices of culturable bacterial and fungal communities derived from mural paintings at different locations in Xu Xianxiu’s Tomb. CH and DR denote the chamber and dromos, respectively. Error bars represent the means ± SD. Significant differences between columns were determined using the *t-*test (*p* ≤ 0.05) and are indicated by dissimilar letters (a, b) above the bars.

The diversity of culturable microbial communities was expressed using the Shannon-Wiener diversity index (H′). In the airborne bacteria samples, the one-way ANOVA showed that there was a significant difference in H′ between different sites (*F* = 3.97, *p* = 0.03; Figure 7A). The H′ values of bacteria in the air of the CH and DR sites were 0.67 ± 0.10 and 0.71 ± 0.16, respectively, both of which were significantly lower than that of the OD site, 1.37 ± 0.02. In the mural samples, the H′ values of the CH and the DR sites were 1.00 ± 0.12 and 0.84 ± 0.46, respectively. The *t-*test showed that there was no significant difference in the bacterial community H′ values between the two sites of the chamber and the dromos at the 95% confidence level (*p* = 0.787; Figure 7B). The results of airborne fungal α-diversity were similar to that of bacteria. The H′ values of bacteria in the air of the CH, DR and OD sites were 1.16 ± 0.17, 1.17 ± 0.11 and 1.29 ± 0.10, respectively (Figure 7C). In the mural samples, the *H*′ values of the CH and the DR sites were 0.71 ± 0.12 and 1.10 ± 0.38, respectively (Figure 7D).

**Figure 8:**
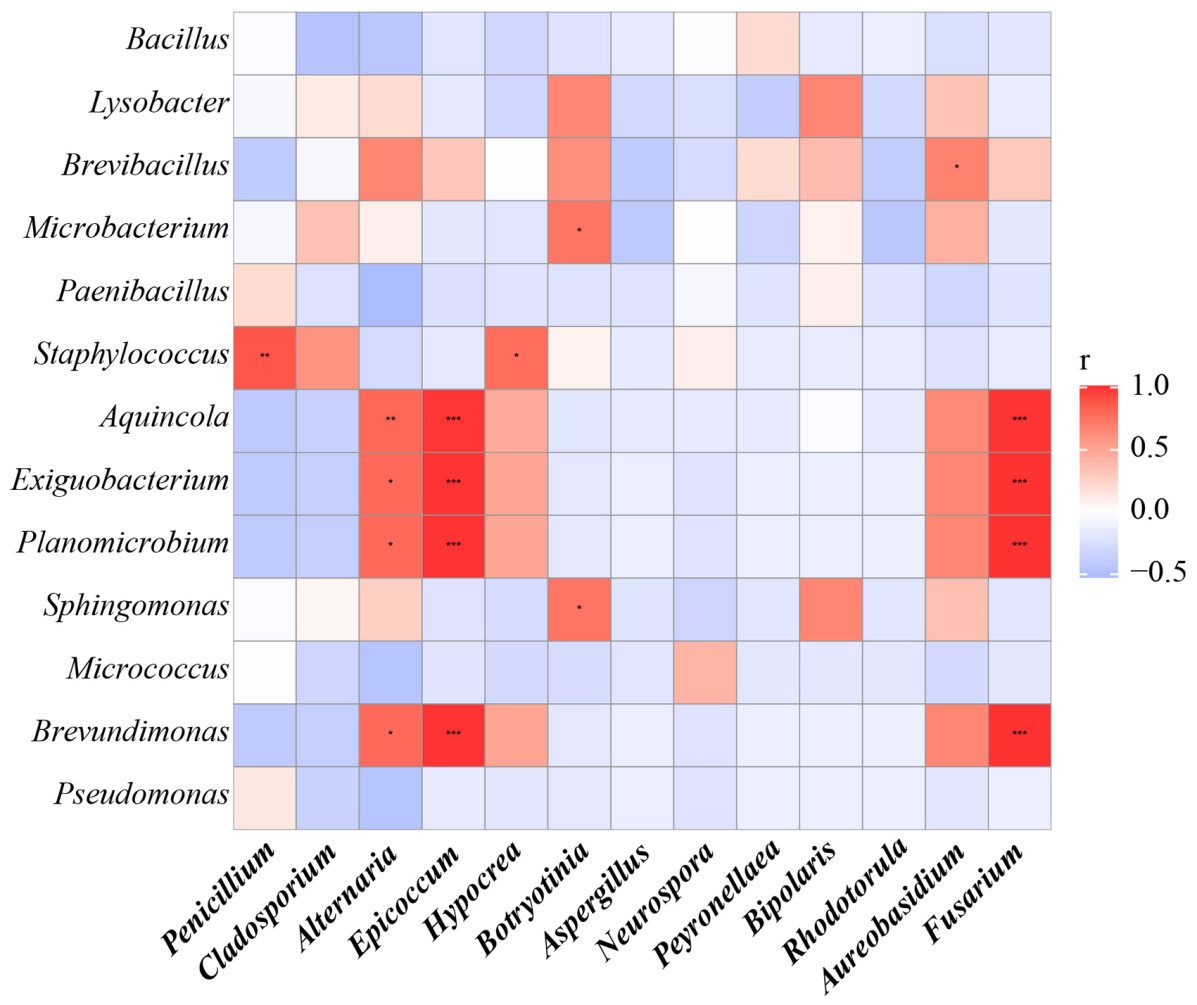
The Spearman correlation analysis between the relative abundance of airborne bacterial and fungal communities at the genus levels. Fungal genera were indicated in bold text. Color key for the correlation values is shown on the right panel inset; positive correlations are in red text, negative correlations are in blue, non-significant correlations are shown in white. * indicates 0.01 < *p* ≤ 0.05, ** indicates 0.001 < *p* ≤ 0.01, and *** indicates *p* ≤ 0.001.

**Figure 9:**
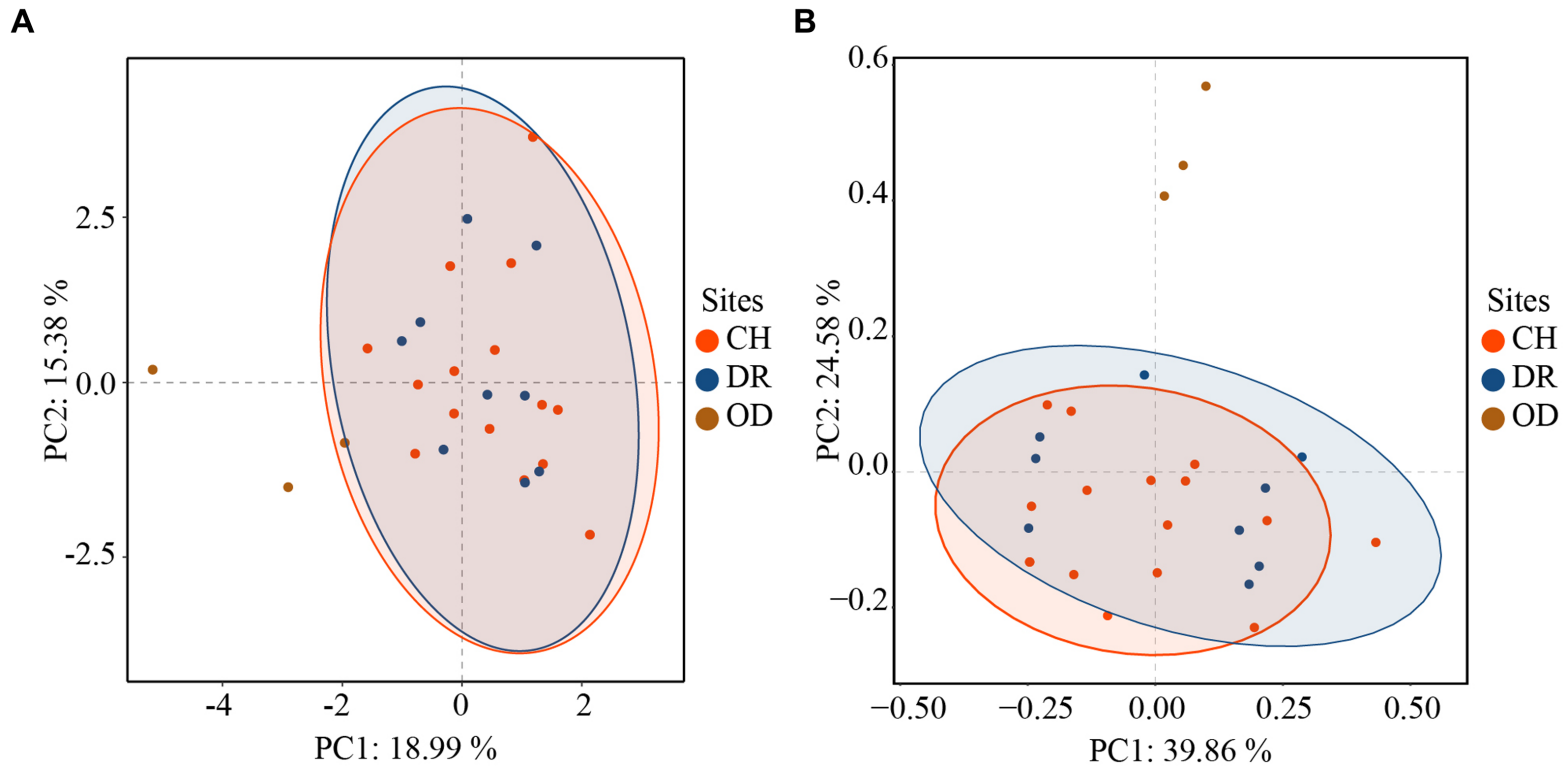
Principal component analysis (PCA) of the airborne bacterial **(A)** communities and airborne fungal **(B)** communities at different sites inside and outside Xu Xianxiu’s Tomb. CH, DR, and OD denote chamber, dromos and outdoors, respectively.

Furthermore, the correlations between bacteria and fungi were analyzed using Spearman analysis (Figure 8). As depicted in the heatmap, *Penicillium*, the dominant genus in the air, only had a significant positive correlation with *Staphylococcus*. There was no significant difference in correlation between *Cladosporium* and all bacterial genus. Both *Alternaria* and *Epicoccum* were positively correlated with *Aquincola*, *Exiguobacterium*, *Planomicrobium* and *Brevibacillus*. In addition, the β-diversities of bacteria and fungi in air environments were used to determine the differences among samples by relative abundance (Figure 9). From the observations of the principal component analysis (PCA), the PC1 axis and PC2 axis explained 18.99 and 15.38%, respectively, of the distributions of airborne bacteria. The bacterial distributions in the CH and DR sites were relatively concentrated and partially overlapped, indicating that the differences in the compositions of the airborne bacterial communities between indoor sites were small. However, the bacterial distribution of the OD site was different from those of the CH and DR sites and did not overlap, indicating that the airborne bacterial community compositions in the outdoors and indoors were quite different. These differences in the structure appearing in bacterial communities were also observed in fungal communities. The fungal communities in the air samples were divided into three groups and were mainly separated by PC1 and PC2, which accounted for 42.17 and 24.58%, respectively, of the amount of community variation. The fungal distributions inside the tomb (including CH and DR) were relatively concentrated and partially overlapped, but they differed greatly from the OD samples. These results suggested that the structure of microbial communities inside and outside the tombs was different, and the composition of bacteria and fungi was closely related.

### 3.3. Comparison of the culturable microorganism communities between airborne and murals in Xu Xianxiu’s Tomb

**Figure 10:**
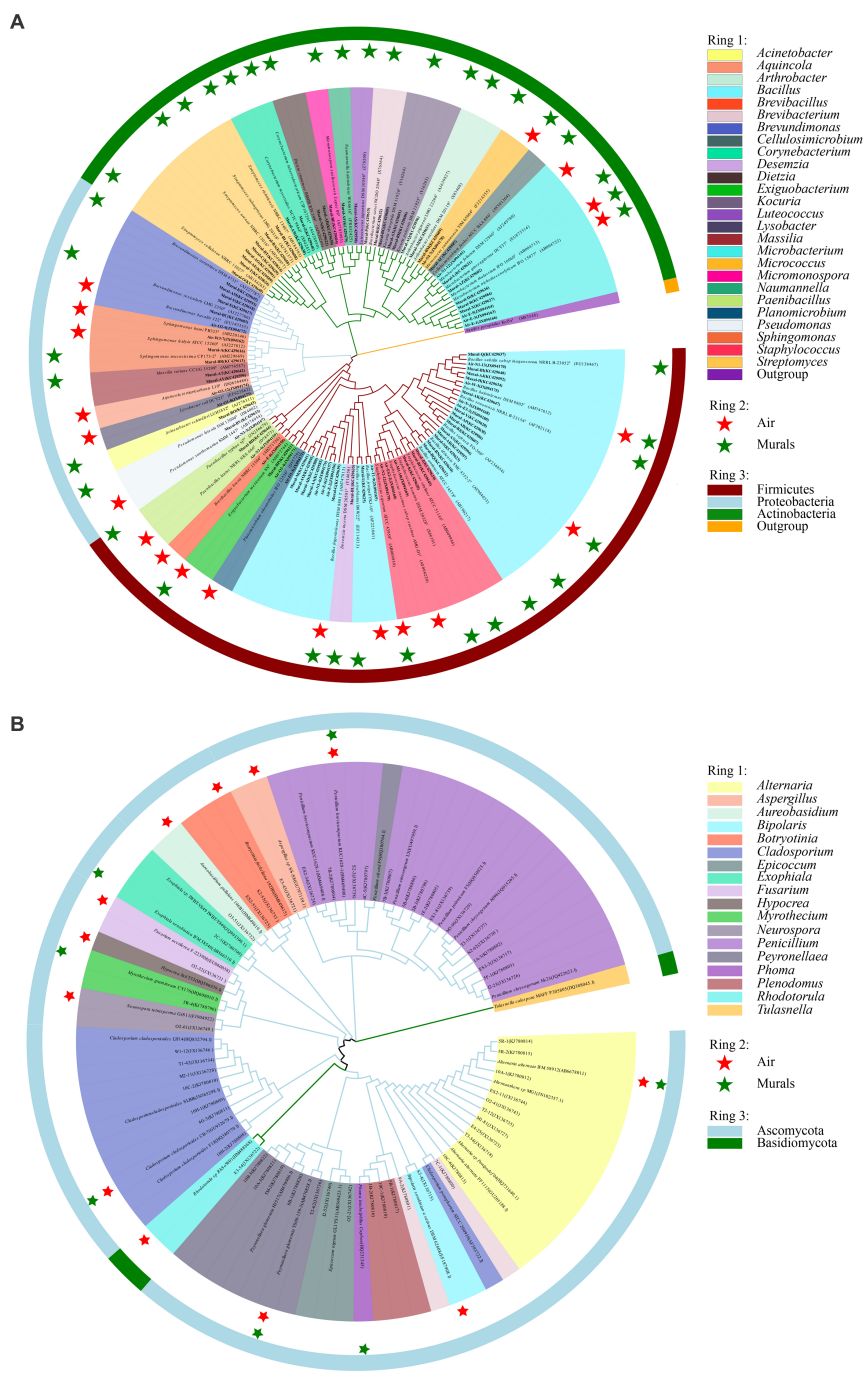
Phylogenetic tree of bacterial **(A)** and fungal **(B)** gene sequences derived from the mural and air environments inside and outside Xu Xianxiu’s Tomb. Air: samples from air environments; Murals: samples from murals. Star indicates that species occurred in the corrsponding sampling site.

There are 6 bacterial species commonly isolated from both the mural and air environments, namely, *Bacillus frigoritolerans*, *Bacillus halotolerans*, *Bacillus safensis*, *Exiguobacterium mexicanum*, *Microbacterium trichothecenolyticum*, and *Micrococcus yunnanensis* (Supplementary Figure S1 and Figure 10A). Among them, *Bacillus frigoritolerans* (74.0%) was the most dominant culturable bacterial group of murals (Supplementary Figure S2). In addition, *Microbacterium trichothecenolyticum* (10.0%), *Bacillus safensis* (0.3%), *Bacillus halotolerans* (0.2%), *Micrococcus yunnanensis* (<0.1%), and *Exiguobacterium mexicanum* (<0.1%) were distributed in mural samples to different degrees. The dominant species in the air samples was *Bacillus safensis* (19%). Moreover, *Microbacterium trichothecenolyticum* (4.4%), *Exiguobacterium mexicanum* (0.9%), *Micrococcus yunnanensis* (0.6%), *Bacillus frigoritolerans* (0.2%), and *Bacillus halotolerans* (0.1%) were all found in mural samples and present in the air samples. Furthermore, there were 4 fungal species commonly isolated from both the mural and air environments, namely, *Alternaria alternata*, *Cladosporium cladosporioides*, *Penicillium brevicompactum*, and *Peyronellaea glomerata* (Figure 10B). Among them, the relative abundance of *Penicillium* in the air environments of the indoor tomb was higher than that outside the tomb. The total relative abundance of *Penicillium* and *Peyronella* in the mural samples accounted for more than 75% of each sampling site (except site I).

### 3.4. Predicted ecological functions of bacteria

**Figure 11:**
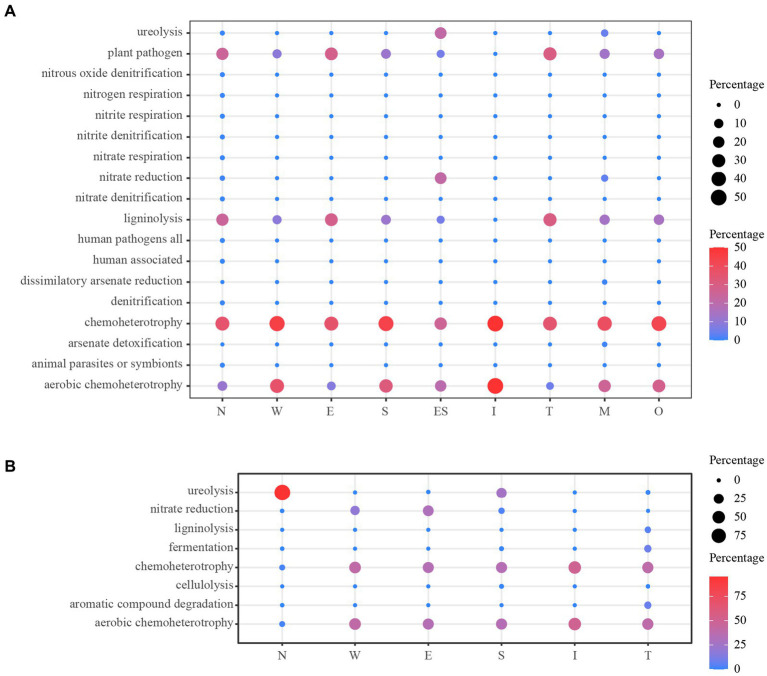
Prediction of the ecological functions for bacterial derived from the air environments **(A)** and mural **(B)**. O: the samples derived from the outdoors (OD) near the entrance of the tomb.

The prediction of the ecological functions of the bacteria derived from the air environments and mural is displayed in Figure 11. The bacterial guilds were 18 identified for air environments and 8 identified for mural. A large amount of the bacterial sequence derived from the air environments was assigned to plant_pathogen, ligninolysis, chemoheterotrophy or aerobic_chemoheterotrophy, and chemoheterotrophy and aerobic_chemoheterotrophy were observed equally in all sample types (100%). The functions plant_pathogen and ligninolysis were rarer in samples taken from the middle of the tomb (i.e., site I). The functions related to ureolysis and nitrate_reduction were predicted; they displayed higher relative proportions in the samples far from the ground (E and M) (Figure 11A). The predicted functional characteristics of the bacterial derived from mural demonstrated a diverse situation. The main bacterial guilds found across all samples were in the following order: chemoheterotrophy, aerobic_chemoheterotrophy, nitrate_reduction, and ureolysis. The first three functional guilds accounted for substantial relative proportions in all sample types, except for samples from N, in which only one guild (ureolysis) showed a higher relative proportion (Figure 11B).

## 4. Discussion

### 4.1. Community differences in airborne microorganisms

Bioaerosols containing airborne microorganisms and their metabolites result in severe aerosol pollution in many indoor environments (Stetzenbach, 2007; Diaz-Alonso et al., 2021). The airborne microorganisms can complete the materials exchange between the environment inside and outside heritage sites through a passive ventilation process. The cells and spores in the aerosol adsorbed onto the surfaces of dust particles, water droplets and small insects can be carried to the surfaces of cultural heritage artifacts in different ways, accelerating the biodeterioration processes, which makes the air microbial communities in heritage sites an important biological indicator of underground heritage preservation (Portillo et al., 2008). Relevant environmental factors, such as relative humidity and temperature, which are important factors that restrict the survival of microorganisms in the air, often determine the relative extent of microbial colonization (Zhang et al., 2021; Amundson et al., 2022). At present, using culture methods to investigate microbial communities in the air has been widely recognized. Among these microbial communities, the taxa with excellent capabilities of spore-forming, as one of the most frequently present types of taxa in aerosol samples, are not only easy to spread in the air but also have a great advantage of being able to grow during the subsequent culture process (Lopez-Miras et al., 2013).

*Lysobacter soli*, the only group of *Lysobacter* found in this study, was originally isolated from the soil of agricultural plantations (Srinivasan et al., 2010). The ground soil of Xu Xianxiu’s Tomb had been used as a field for a pear orchard for many years. Similar soil types may lead to the same composition of organic matter. The accumulation of nutrients that volatilize from the soil into the air of the tomb for long time results in the selective growth of the same dominant taxa. In addition, *Bacillus* has always been one of the common taxa in cultural heritage sites and is widely distributed all over the world, which has a beneficial characteristic of spore-forming that enables it to adapt to extreme environments to a greater extent (Yang and Sun, 2020).

The dominant fungal genera identified in this study (such as *Penicillium* sp., *Alternaria* sp., *Cladosporium* sp., and *Aspergillus* sp.) were widely distributed in air, soil and indoor environments (St-Germain and Summerbell, 2003). With the exception of *Exophiala xenobiotica*, *Rhodotorula* sp. and a few uncommon genera, most fungi were the species with high reproductive capacity of spore-producing and can be cultured. The dominant species found in Xu Xianxiu’s tomb were consistent with most of the investigation on microorganisms in tombs and caves, but the diversity was not exactly the same. The fungi isolated and identified from Xu’s tomb mainly included *Penicillium* sp., *Cladosporium* sp., *Aspergillus* sp. and the remaining 14 genera. There were 16 genera that belonged to ascomycetes, many species of which were widely distributed around the world (Gaylarde and Gaylarde, 2000).

The indoor air bacterial concentration of a university detected by Lou et al. is up to 700–2000 CFU/m^3^, which is believed to be related to population aggregation to a certain degree (Lou et al., 2012). In the Qin Shihuang Terracotta Warriors and Horses Museum, the fungal concentration once reached 25,000–45,000 CFU/m^3^ during the peak period of the tour, which is enough to show that human activities have a certain impact on the air microorganisms in closed environments (Chen et al., 2010). By monitoring the air bacterial communities of the closed, semiclosed and completely open caves in the Mogao Grottoes, the researchers found that the air contains a high bacterial concentration in closed and completely open caves, while the bacterial concentration is lowest approximately 500 CFU/m^3^ in the semienclosed caves (Wang et al., 2012), which is similar to this study. Probably due to the passing of a few heritage conservation staff members of Xu Xianxiu’s Tomb, making the air circulation stronger than that when the tomb was completely closed.

In this study, the airborne bacterial and fungal community structures outside and inside the tomb were significantly different. PCA showed that the bacterial and fungal distributions of the samples collected from indoor sites were concentrated and overlapped, while those of the samples collected from outdoor sites were different from those of the indoor sites. At the same time, the Shannon-Wiener indices of the airborne bacteria and fungi in the indoor sites of Xu Xianxiu’s Tomb was significantly lower than those in the outdoor sites, and the airborne bacterial and fungal diversity indices in the dromos were slightly higher than those inside the tomb. The differences of the structure and diversity of microbial communities in the air inside and outside the tomb are closely related to the plastic greenhouses covered above the tomb. The dissemination of nutrients in aerosols usually relies on passive gas exchange or ventilation systems (Myers et al., 2021), similar to the Etruscan catacomb (Monte and Ferrari, 1993). The greenhouse in this study can play a role in restricting the gas inflow and weakening the air exchange between the inside and outside of the tomb so that a buffer zone forms in the process of air spreading from the outside air to the inside air, which also explains why the air microbial diversity indices of the dromos were slightly higher than those of the chamber. At the same time, the disturbance of the dust deposited at the sampling sites in the tomb during the sampling process can also lead to the temporary resuspension of some microbial in the air, which explained why there were no significant differences in the overall diversity of the air bacterial community among the sites in the indoor environment of the caves (Wang et al., 2010).

In addition, the relative humidities and temperatures in the internal and external environments of the catacomb are quite different. The previous study has also shown that microorganisms colonize the surfaces of historical heritage artifacts exposed to changing environmental conditions, such as temperature, relative humidity, pH and sunlight (Caneva et al., 2021), and the microbial outbreaks were primarily attributed to the excessive air humidity of the inside and outside sampling sites (He et al., 2021, 2022a). The temperature outside the greenhouse can reach 35°C in summer, which is the most suitable growth temperature for most bacterial groups, while the maximum temperature inside the greenhouse is only 25°C. The reduction in temperature can effectively control the growth of most microbial groups, suggesting that the erection of greenhouses had positive significance for maintaining the stability of the internal microenvironment of the tomb. Furthermore, Wu et al. (2016) found that the relative humidity in the lower part of the tomb passage was always high, which was a key environmental factor that induced fungal mildew on the murals of Xu Xianxiu’s tomb. Therefore, the protective shed can isolate and buffer the particulate matter and microorganism in the external environment to a certain extent.

### 4.2. Microorganisms community differences in murals

As an important component of cultural heritage, murals have irreproducible aesthetic and historical value, of which the physicochemical properties often induce different types of microbial colonization (Krakova et al., 2015; Duan et al., 2021). Heterotrophic microorganisms can use the sufficient organic nutrients provided by autotrophic microorganisms to colonize mural surfaces (Karpovichtate and Rebrikova, 1991). The most common heterotrophic bacterial groups in the murals of cultural heritage sites are *Arthrobacter*, *Bacillus*, *Paenibacillus*, *Flavobacterium*, *Pseudomonas*, *Micrococcus*, *Staphylococcus*, *Nocardia*, and *Mycobacterium* (Elhagrassy, 2018; He et al., 2021; Suphaphimol et al., 2022).

The culturable bacterial groups in the murals of Xu Xianxiu’s Tomb are mainly *Firmicutes*, *Actinobacteria* and *Proteobacteria*. The abundance of *Firmicutes* on the mural surfaces is similar to the study by Pangallo et al. (2015), in which, through the cultivated method, they also found that *Firmicutes* isolated from the surface of a statue was abundant (Pangallo et al., 2015). *Bacillus* (81.4%), the most dominant group of culturable bacteria on the mural painting surfaces of Xu Xianxiu’s Tomb, was also found to be enriched on the surfaces of the murals and had a proportion of 67% through isolation and cultivation in 2012 (Pangallo et al., 2012). Researchers have pointed out that the higher relative abundance of *Bacillus* isolated by traditional culture methods has a strong relationship with its unique capability of spore formation, which can support its rapid growth in media (Gurtner et al., 2000). Additionally, there was a significant difference in the concentrations of culturable bacteria on the mural painting surfaces between the chamber and the dromos because the dominant groups of the mural bacterial communities have different distributions between the chamber and the dromos, resulting in the different distributions of the concentrations and structures of bacterial communities at the two sites (Ciferri, 1999; Saiz-Jimenez et al., 2012).

In our study, most of the fungi isolated from the murals belonged to the *Penicillium, Cladosporium* and *Aspergillus* genera. *Penicillium* was a common fungus that can be isolated from the mural surface in tomb. Most species with strong reproductive capacity isolated from the mural surface in tomb fungi can be cultured, most of which could produce spores and mycelia except some rare species (such as *Exophiala xenobiotica* and *Rhodotorula* sp.) (Pangallo et al., 2012; He et al., 2022b). Studies had shown that these common fungal genera can hydrolyze proteins in this special environment (Pangallo et al., 2012; Unkovic et al., 2018). It was well known that murals contained mass protein-based adhesives, which will be destroyed by the long-term colonization of these fungi and lead to a microbial biodeterioration (Unkovic et al., 2018). In addition, *Penicillium*, *Cladosporium* and *Aspergillus* can produce malic acid in the environment, which may contribute to the proliferation of some heterotrophic bacteria (Peleg et al., 1988). Furthermore, these organic acids can further stimulate the growth of fungi and bacteria, and eventually lead to a large number of microbial colonization on the surface of the murals (Vasanthakumar et al., 2013). By collecting the mural samples with whitish moldy necrosis of Xu Xianxiu’s tomb, Wu et al. (2021a) found that the main culturable fungus that caused mildew on the corridor wall of the tomb was *Parengyodontium album*, which was the dominant cultivated fungi.

### 4.3. Correlation analysis of microorganism communities in the air and on mural paintings

The relationship of the microbial communities between the air and murals is inseparable. Dust, dirt and the remains of animals and plants will persist on the surfaces of the murals through the exchange of substances between the air and the murals, providing a material basis for the colonization of microorganisms (Ciferri, 1999; Saiz-Jimenez and Gonzalez, 2007). The microbial community in the air around the murals can also be directly adsorbed on the mural painting surfaces, resulting in large-scale colonization with the help of minerals and organic nutrients. Moreover, the microorganisms grown inside the mural paintings can also be dispersed into the surrounding air through ventilation facilities or anthropogenic disturbance (Sanchez-Moral et al., 2005; Pangallo et al., 2012).

To study the relationship between the air and the mural microbial communities in this tomb, we compared the coexisting bacterial and fungal strains by the culture method and found that there were 6 coexisting bacterial species in the air and murals in Xu Xianxiu’s Tomb, among which *Bacillus frigoritolerans* was the most dominant bacterial group on the mural surfaces, with a relative abundance of 74.0%, while it only accounted for 0.2% of the airborne bacterial community. This indicates that this group may be the original bacterial group in the murals, and the primary source of its appearance in the air is its passive diffusion from the murals. However, *Bacillus safensis*, which accounted for only 0.3% of the culturable bacteria in the murals, was the dominant group in the air (19%), suggesting that airborne transmission was the main source of this group colonizing the mural surfaces. In addition, the appearance of *Microbacterium trichothecenolyticum*, *Bacillus safensis*, *Bacillus halotolerans*, *Micrococcus yunnanensis*, and *Exiguobacterium mexicanum* in both murals and air indicated that there was a close internal relationship between the mural and airborne bacterial communities (Saiz-Jimenez and Gonzalez, 2007). There were only 9 genera of mural fungi, but four of them were not present in the air samples. However, the proportion of genera unique to the murals was low, suggesting that a large part of the influence on the murals come from the airborne fungi, but a small part come from other disturbances. For example, when the tomb of Tutankhamun in Egypt was opened, there were many brown fungal spots were found on the mural surface, which microbiologists identified as dead after more than 2,000 years of isolation (Fahd, 1994; Vasanthakumar et al., 2013). Secondly, the microbial diversity on the mural paintings usually had a spatio-temporal dynamic succession process (Ciferri, 1999; Bastian et al., 2010). Changes in the microenvironment or nutrient sources in the tomb will lead to new changes in the diversity of disease microorganisms. Remarkably, He et al. (2022b) found that the surface active microorganism concentration of tomb mural was significantly higher than that of tomb ramp, which may be caused by human activities.

Furthermore, the prediction of the ecological functions of the bacteria derived from the air environments and mural was carried out to further compare the differences of functional metabolism between bacteria from different sample types. The bacterial guilds for air environments were more than that for mural indicating the more abundant metabolic function. Conspicuously, the functions related to human could be found in air environments, but not in mural; this may have been a result of effective protection measures which irregularly restricted tourist activities. As air is an important medium for the transmission and diffusion of microorganisms, the prevention and control of airborne microorganisms has always been an important part of cultural heritage protection (Porca et al., 2011; Saiz-Jimenez, 2012). Air is the material communication channel between the internal and external environments of cultural heritage and is also related to the colonization of microbial communities on the surfaces of artefacts inside cultural heritage. Therefore, the demonstration of the relationship between the air and mural microorganisms is not only crucial to the scientific understanding of the existence of microorganisms in murals and even the entire cultural site environment but can also provide a scientific reference for the rational protection of murals.

## 5. Conclusion

In summary, this study systematically reported the concentrations, diversities, and community compositions of microorganisms in the air and mural painting surfaces inside and outside Xu Xianxiu’s Tomb. The culturable bacteria belonged to *Bacillus*, *Microbacterium*, *Lysobacter*, and *Arthrobacter*. And the most fungal species belonged to the *Penicillium*, *Cladosporium*, and *Aspergillus* genera. The composition and structure of airborne bacteria and fungi outside the tomb were both significantly different from those inside the tomb. The variation trends of airborne bacterial and fungal concentrations at different sampling sites were remarkably similar. The prediction of the ecological functions of the bacteria revealed that chemoheterotrophy or aerobic_chemoheterotrophy accounted for substantial relative proportions in all sample types. Temperature and humidity can affect the selective colonization of microbia, and the interference of human activities on microbia in the process of mural biodeterioration cannot be ignored. In the process of protecting the murals in the tomb, the adjustments by the greenhouse of the temperature, humidity and air circulation inside and outside were beneficial for controlling the massive growth of bacteria and fungi on wall paintings. In the future protection work, attention should also be given to the roles of ventilation systems and artificial material inflow in the microenvironment of the tomb. Consequently, strict control of the environmental factors of indoor catacombs we recommend even at restoration stages for better conservation.

## Data availability statement

The datasets presented in this study can be found in online repositories. The names of the repository/repositories and accession number(s) can be found in the article/supplementary material.

## Author contributions

JL: Writing – original draft, Conceptualization, Formal analysis, Investigation, Methodology, Software. FW: Writing – original draft, Conceptualization, Formal analysis, Investigation, Methodology, Software. TX: Writing – review & editing, Data curation, Investigation, Methodology. WM: Data curation, Writing – original draft. DH: Writing – review & editing, Investigation, Visualization. QZ: Writing – review & editing, Resources, Supervision. WW: Writing – review & editing, Software, Validation. YD: Writing – review & editing, Software, Validation. TT: Writing – review & editing, Conceptualization, Funding acquisition, Resources, Supervision. HF: Writing – review & editing, Conceptualization, Funding acquisition, Resources, Supervision.

## Footnotes

^1^ http://blast.ncbi.nlm.nih.gov/Blast.cgi

^2^ http://cran.r-project.org/

^3^ https://pages.uoregon.edu/slouca/LoucaLab/archive/FAPROTAX/lib/php/index.php
