# Supplementary material for: Differences of airborne and mural microorganisms in a 1,500-year-old Xu Xianxiu’s Tomb, Taiyuan, China
Source: Front Microbiol. 2023 Oct 25;14:1253461. doi: 10.3389/fmicb.2023.1253461 (PMC10635417; doi:10.3389/fmicb.2023.1253461)

Differences of airborne and mural microorganisms in a 1500-year-old Xu Xianxiu's Tomb, Taiyuan, China

Jiangyun Liu[1‡], Fasi Wu[2, 3‡], Ting Xiang[4], Wenxia Ma[4], Dongpeng He[2, 3], Qi Zhang[4], Wanfu Wang[2, 3], Yulong Duan[5], Tian Tian[1*], Huyuan Feng[4*]

[1] School of Public Health, Lanzhou University, Lanzhou, Gansu, 730000, The People's Republic of China
[2] National Research Center for Conservation of Ancient Wall Paintings and Earthen Sites, Department of Conservation Research, Dunhuang Academy, Dunhuang, Gansu, 736200, The People's Republic of China
[3] Gansu Provincial Research Center for Conservation of Dunhuang Cultural Heritage, Dunhuang, Gansu, 736200, The People's Republic of China
[4] MOE Key Laboratory of Cell Activities and Stress Adaptations, Centre for Grassland Microbiome, School of Life Sciences, Lanzhou University, Lanzhou, Gansu, 730000, The People's Republic of China
[5] Northwest Institute of Eco-Environment and Resources, Chinese Academy of Sciences, Lanzhou, Gansu, 730000, The People's Republic of China

[‡] Co-first author, contributed equally to this work;
*Corresponding author: tiant@lzu.edu.cn (T.Tian); wufs@dha.ac.cn (F.Wu); fenghy@lzu.edu.cn (H. Feng);

Supplementary Figure S1. The Venn diagram of bacteria isolated from air and murals of Xu Xianxiu's Tomb.

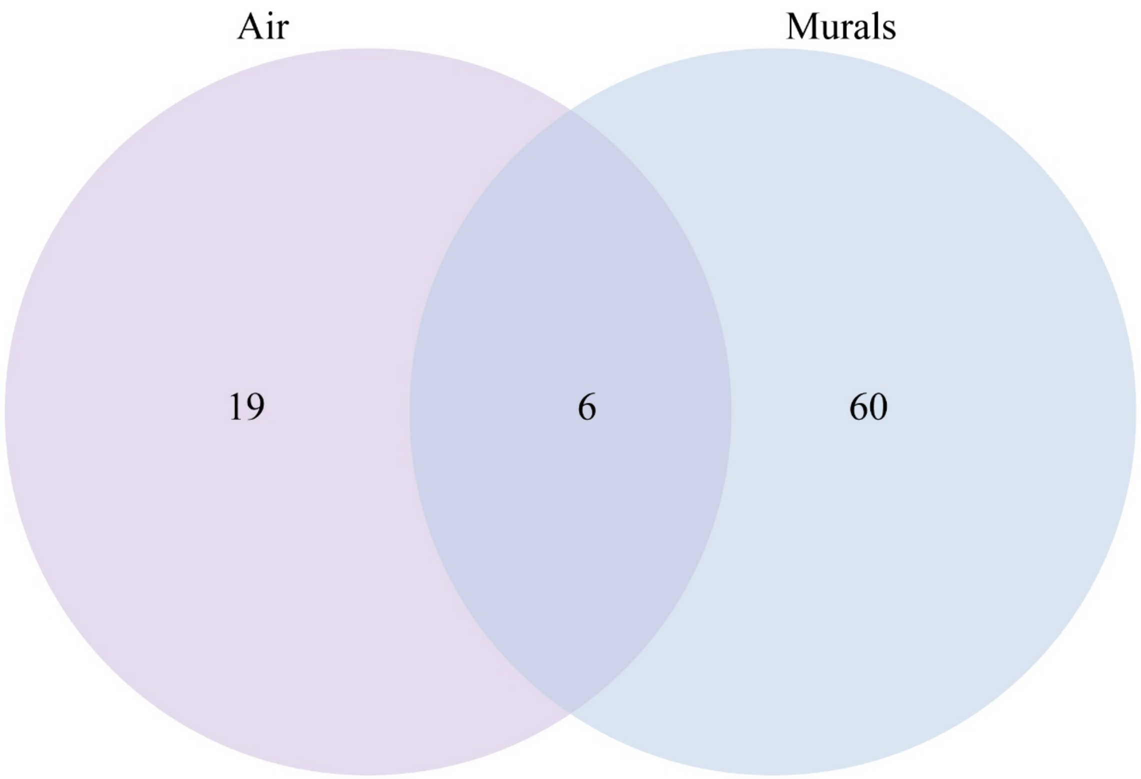

Supplementary Figure S2. The bacterial species commonly isolated from both the air (a) and mural (b) environments.

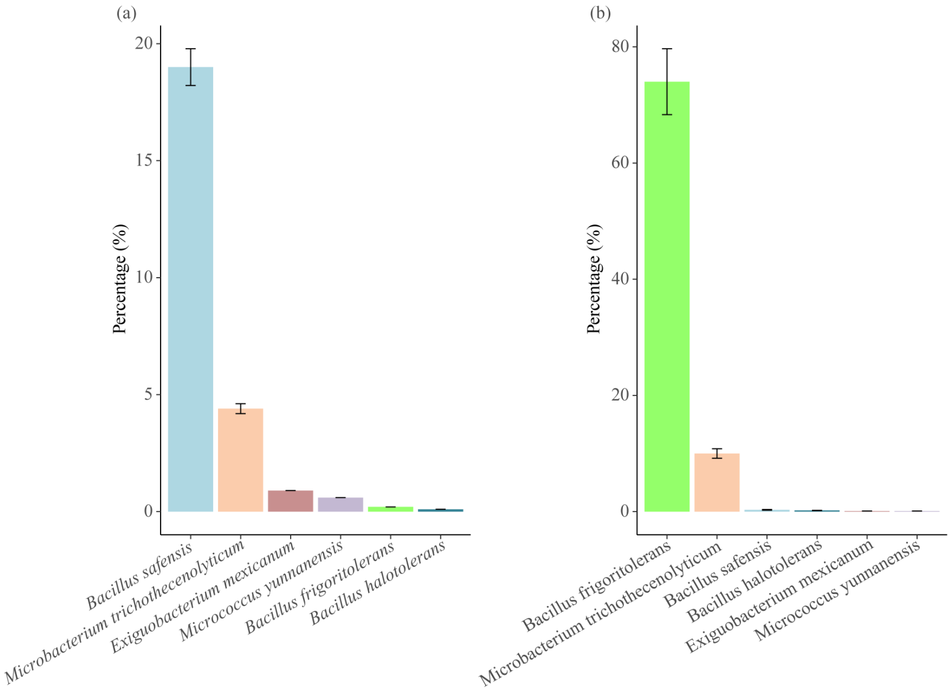

Supplement: Supplementary file 1 [file Image_1.pdf]
